# Comparison of STONE, Guy’s Stone, and Seoul National University Renal Stone Complexity (S-ReSC) Scoring Systems for PCNL Monotherapy in Staghorn Stones

**DOI:** 10.5152/tud.2025.24128

**Published:** 2025-04-04

**Authors:** Santhosh Srinivasan, Sharanya Padma, Abdul Azeez Ambalath, Poulose Chally, Pankaj Bhirud

**Affiliations:** 1 Department of Urology, Sri Devaraj Urs Medical College, Karnataka, India; 2 Department of Dermatology, Manipal Hospitals, Karnataka, India; 3 Department of Urology, Baby Memorial Hospital, Kerala, India; 4 Department of Urology, Metromed Institute of Advanced Urology and Renal Transplantation, Kerala, India

**Keywords:** Staghorn stones, percutaneous nephrolithotomy, STONE score, Guy’s stone score, S-ReSC score

## Abstract

**Objective:**

Accurate evaluation of staghorn stones is crucial for predicting the likelihood of achieving stone-free status through percutaneous nephrolithotomy. Scoring systems such as the Stone size, Tract length, Obstruction, Number of calyces, Essence of stone density (STONE) score, Guy’s Stone score, and Seoul National University Renal Stone Complexity (S-ReSC) score have been developed to quantify stone complexity and guide clinical decision-making.

**Methods:**

This was a prospective comparative study conducted with 52 staghorn calculi patients. Grading of the stone was done by using 3 scoring systems. An imaging study using ultrasound kidneys, ureters, and bladder was performed for early detection of remnant fragments to determine stone-free rate on postoperative day 4. Postoperative complications were categorized by using Clavien–Dindo classification system. Receiver operating characteristic curves were constructed to evaluate the predictive value of 3 stone criteria on the stone-free rate.

**Results:**

According to the Guy’s Stone score and S-ReSC score systems, all patients with grade IV and high complexity stones had residual stones by the end of POD 4. In contrast, the STONE criteria reported that 11.1% of high complexity stones were stone-free. All 3 stone scoring systems indicated a significant increase in the occurrence of complications with increasing stone complexity. The S-ReSC scoring system exhibited the highest AUC of 0.831, indicating it has superior predictive performance compared to Guy’s Stone criteria (AUC: 0.790) and the STONE criteria (AUC: 0.765).

**Conclusions:**

Among the STONE, Guy’s Stone, and S-ReSC scoring systems, the S-ReSC scoring system has proven to be the most effective for assessing both SFR and complications.

## Introduction

Staghorn calculi (SC) are large stones that occupy the pelvicalyceal system (PCS). These stones fill the renal pelvis and extend into several or all of the calyces. A stone that branches but occupies only part of the collecting system is referred to as a partial staghorn calculus, while a complete staghorn calculus is one that occupies the entire collecting system.^1^ Staghorn stones can cause significant morbidity if not effectively treated, including recurrent infections, impaired renal function, and potentially irreversible kidney damage.^2^

The primary goal in treating staghorn calculi is complete removal. Treatment options include percutaneous nephrolithotomy (PCNL), shock wave lithotripsy (SWL), retrograde intrarenal surgery (RIRS), ureteroscopy, laparoscopy, open surgery, or a combination of these methods.^3^ Treatment success depends on factors such as stone complexity and surgeon expertise, with multiple surgeries sometimes necessary. Percutaneous nephrolithotomy remains the gold standard for most patients due to its effectiveness and low morbidity.^4^ Advancements in minimally invasive techniques, miniaturized equipment, and improved fragmentation tools have further enhanced the surgical management of these complex stones. In an initial case series documenting PCNL outcomes for staghorn stones, the authors observed complete stone clearance rates of 98.5% for partial staghorn stones and 71% for complete staghorn stones.^5^ However, due to the complexity of these stones, a meticulous preoperative assessment is necessary to optimize surgical outcomes.

Accurate evaluation of staghorn stones is crucial for predicting the likelihood of achieving stone-free status through PCNL. Scoring systems such as the STONE score, Guy’s Stone score, and Seoul National University Renal Stone Complexity (S-ReSC) score have been developed to quantify stone complexity and guide clinical decision-making.^5-10^ Each of these scoring systems incorporates different parameters and methodologies to assess the stone burden and anatomical challenges associated with staghorn stones.

This study seeks to compare the efficacy and predictive value of the STONE, Guy’s Stone, and S-ReSC scoring systems in the context of PCNL monotherapy for staghorn stones. By analyzing their correlations with surgical outcomes, complication rates, and stone-free status, researchers aim to determine which scoring system provides the most accurate and practical guidance for urologists, ultimately improving patient care and outcomes in the management of complex renal stones.

## Materials and Methods

This was a prospective comparative study conducted at Department of Urology at Baby Memorial Hospital, Kozhikode, India between May 2014 and April 2016. Patients aged 20 to 80 years with radiologically confirmed staghorn calculi, classified as either partial or complete, and those who underwent PCNL as the primary treatment modality were included in the study. Patients with active urinary tract infection, uncontrolled diabetes mellitus, bleeding disorders, and renal dysfunction with serum creatinine levels above 1.5 mg/dL were excluded from the study.

**Sample size**: The following formula was used to estimate the sample size:



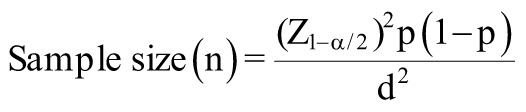



In this calculation, Z_1-α/2_ was 1.96 (from the Z table) at a 95% level of significance. The proportion of patients who achieved a stone-free rate after PCNL (p) was 75%, as reported in a Karnataka-based study by Deole S et al.^11^ The relative error or precision was set at 17% of p. Based on these inputs, the calculated sample size (n) was 45. Adjusting for a 10% loss to follow-up, the effective sample size was increased to 52. Consequently, this study included a total of 52 patients with staghorn calculi treated at Department of Urology at Baby Memorial Hospital, Kozhikode, India, for comparing the STONE, Guy’s Stone, and S-ReSC scoring systems.

### Sampling Technique

Consecutive enrollment of patients who underwent PCNL was done until the desired sample size was reached.

### Study Procedure

#### Preoperative Investigation:

All the selected patients were subjected to routine blood, biochemical, and urine examinations with computed tomography kidneys, ureters, bladder (CT KUB) split bolus protocol preoperatively.^12^ Then grading of the stone was done by using 3 scoring systems in all the selected patients. The S.T.O.N.E criteria scoring was based on stone size, tract length, obstruction, number of calyces involved, and essence of stone density.^6,7^ In Guy’s Stone score system, grade 1 is assigned for a solitary stone located in the pelvis or a single calyx; grade 2 is for multiple stones in 1 kidney or a single stone with anatomical abnormalities; grade 3 applies to stones in multiple locations or the presence of anatomical issues that complicate PCNL; and grade 4 is for staghorn stones or any stone in a solitary kidney with abnormal anatomy.^8,9^ The S-ReSC Score was determined by tallying the number of involved sites, without considering the size or quantity of stones. The sites are as follows: the first site is the renal pelvis; the second and third sites are in the superior and inferior major calyceal groups, including the infundibulum; the fourth and fifth sites are in the superior minor calyceal system; the sixth and seventh sites are in the middle calyceal system; and the eighth and ninth sites are in the inferior minor calyceal system. Each site is assigned 1 point, leading to a maximum possible score of 9 points. Later, based on total scores, stones were categorized into low complexity (total score of 1 to 2), medium complexity (total score of 3 to 4), and high complexity (total score of 5 to 9).^10^

### Intervention

All patients underwent PCNL performed by the same surgeon under general anesthesia. Initially, they were given a loading dose of antibiotics. The procedure began with the patients positioned in lithotomy for cystoscopy, during which a 16-French Foley catheter was inserted into the affected ureter to facilitate catheterization. Following this, the patients were repositioned into the prone position to allow access to the kidneys. A retrograde pyelogram was performed by injecting contrast dye into the ureter through the previously placed catheter, allowing visualization of the kidney’s anatomy and the pelvicalyceal system. This helped the surgeon to determine the most suitable calyx for access. Under fluoroscopic guidance, the surgeon identified the target calyx and made a fine needle puncture through the skin into the renal parenchyma, carefully avoiding nearby structures like blood vessels. If the stones were large or spread across multiple calyces, additional punctures were made to ensure adequate access. After the puncture, the tract was dilated using a series of Alken metallic co-axial dilators. The dilators were progressively inserted to gradually widen the puncture site and create a passage large enough for the nephroscope and other instruments. The tract was dilated to a 30-French size, which provided sufficient space for the nephroscope and retrieval of stone fragments. Once the tract was dilated, an Amplatz sheath (a large-bore tube) was inserted into the tract. The sheath provided a stable working channel for instruments and helped maintain access to the kidney throughout the procedure. A nephroscope was introduced through the Amplatz sheath. This flexible instrument allowed direct visualization of the kidney’s interior and stone location. Once visualized, the stones were fragmented using a pneumatic lithotripter. After stone fragmentation, a 14/16-French nephrostomy tube was inserted through the sheath into the kidney to drain any residual urine, debris, or fragments. The tube was secured in place with an Ethilon suture to prevent displacement. In cases where there was a risk of residual stone fragments migrating into the ureter, a Double-J (DJ) stent was placed to ensure proper drainage and prevent obstruction. A total of 3 patients required the placement of a DJ stent.

### Post-operative Investigation

Initial imaging using either x-ray KUB or ultrasound KUB was used for early detection of remnant fragments to determine stone-free rate (SFR) on postoperative day (POD) 4. The choice of imaging modality for each case depended on the opacity of the stone. X-ray was typically selected for radiopaque stones, which are easily visible on x-rays, while ultrasound was preferred for radiolucent stones or when minimizing radiation exposure was a priority. Follow-up imaging using CT KUB was performed 30 days postoperatively to assess stone clearance. Based on clinical practice and supporting literature, the principal investigator, along with other investigators, decided a 30-day follow-up period, as this timeframe is associated with the resolution of fragments or significant reduction in stone burden in most patients with staghorn stones. X-ray and ultrasound were selected on POD 4 to assess stone clearance because of their cost-effectiveness, non-invasiveness, and ability to accurately detect residual stones with minimal radiation exposure. Additionally, these imaging techniques offer the benefit of identifying complications such as bleeding, hydronephrosis, or urinary tract obstruction. A CT scan at 30 days was employed for evaluating stone-free status, as it can detect even small residual fragments that may not be visible on x-ray or ultrasound.

### Outcome Measures

#### Stone-Free Rate:

Defined as the absence of residual stones or the presence of clinically insignificant residual fragments (CIRFs). Clinically insignificant residual fragments are described as asymptomatic, non-infectious, and non-obstructive stone fragments (≤ 4 mm).^13,14^

#### Complication Rates:

Complications were categorized by using Clavien–Dindo classification system.^15^ This system classifies complications into 5 grades, in which grade I consists of minor issues that do not require intervention; grade II includes complications that need specific pharmacological treatment; grade III encompasses major complications, with Ggade IIIa managed through non-invasive methods like percutaneous drainage or endoscopy, and grade IIIb requiring invasive surgical procedures such as laparotomy; grade IV covers life-threatening complications, with grade IVa involving single organ failure necessitating ICU care, grade IVb involving multiple organ failures requiring extensive ICU management; and grade V refers to complications that result in patient death.

### Ethical Clearance

The study was granted ethical approval by the Institutional Ethics Committee (IEC) of Baby Memorial Hospital, Kozhikode, India, with reference number BMH/IEC/21/2024, dated August 12, 2024.

### Informed Patients Consent

Informed consent was secured from all the study participants and their relatives in their native language, covering both participation in the study and the publication of the results.

### Statistical Analysis

Data collected from the participants were entered into Microsoft Excel, and analysis was done using Statistical Package for Social Sciences (SPSS) Statistics version 25.0 (IBM SPSS Corp.; Armonk, NY, USA). Categorical data were presented in proportions, and associations were examined with chi-square tests. Receiver operating characteristic (ROC) curve analysis was conducted to evaluate the predictive accuracy of the 3 scoring systems for the stone-free rate after PCNL, by estimating the area under the curve (AUC) with 95% confidence intervals (CI). DeLong test was conducted to study the difference between AUCs of individual scoring systems. A *P*-value of less than .05 was considered statistically significant for all analyses.

## Results

Demographic and clinical characteristics of the patients having staghorn calculi was reported in Table 1. The majority of patients were between 41 and 60 years of age [n = 34; 65.4%], with a mean age of 51.5 (±11.16) years. Predominantly, they were male [n = 35; 67.3%], had normal body mass index (BMI) [n = 30; 57.7%], and the stones were primarily located on the right side [n = 27; 51.9%]. All patients reported abdominal pain [n = 52; 100%], while 13 (25%) had hematuria and 5 (9.6%) had fever. Around 24 (46.1%) had stone sizes less than 400 mm^2^, 16 (30.8%) had sizes between 400 and 799 mm^2^, 8 (15.4%) had sizes between 800 and 1599 mm^2^, and 4 (7.7%) had stone sizes of 1600 mm^2^ and above. A vast majority of patients, nearly 51 (98.1%), had tract lengths of less than 100 mm, with only 1 (1.9%) having a tract length of 100 mm or more. Obstruction was seen in 26 (50%) patients. Additionally, 34 (65.4%) had stones occupying 1 or 2 calyces, 12 (23.1%) had stones occupying 3 calyces, and the remaining 6 (11.5%) had complete staghorn formation. Furthermore, nearly 5 (9.6%) had stone densities of 500 Hounsfield units (HU) or less, whereas 21 (78.9%) patients had densities between 501 and 1000 HU, and 6 (11.5%) had densities above 1000 HU.

**Figure 1.**
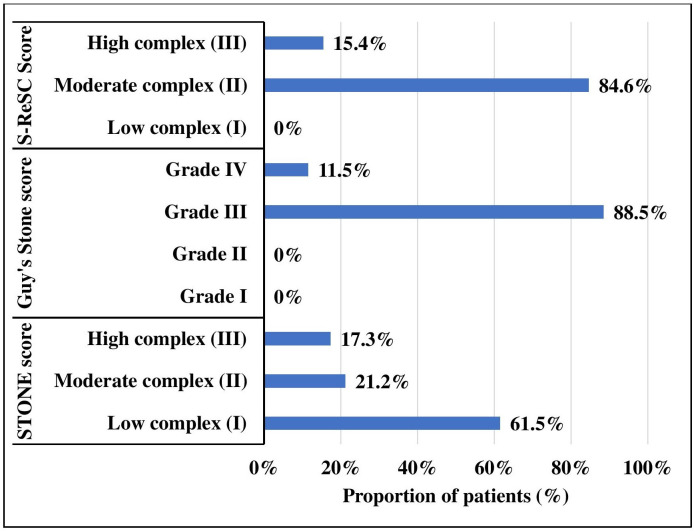
Categorization of staghorn stone based on STONE, Guy’s stone, and S-ReSC scoring systems.

According to the STONE criteria, staghorn stones were classified into low complexity in 32 (61.5%) patients, moderate complexity in 11 (21.2%) patients, and high complexity in 9 (17.3%) patients. In contrast, Guy’s Stone score categorized 46 (88.5%) patients as grade III and 6 (11.5%) patients as grade IV. The S-ReSC score system classified 44 (84.6%) patients as having moderately complex stones and 8 (15.4%) patients as having highly complex stones (Figure 1). On the fourth post-op day, residual stones were present in 17 (32.7%) patients, while 35 (67.3%) were stone-free. When comparing the different stone criteria with stone-free status (Table 2), it was found that, according to the Guy’s Stone score and S-ReSC Score systems, all patients with grade IV and high complexity stones respectively had residual stones by the end of POD 4. In contrast, the STONE criteria reported that 11.1% of high complexity stones were stone-free. Across all 3 criteria, as stone complexity increased, the likelihood of residual stones also significantly increased.

According to the Clavien–Dindo classification of complications (Table 3), 39 (75%) patients experienced no complications, 7 (13.5%) patients had grade I complications, and 6 (11.5%) patients had grade II complications. All 3 stone scoring systems indicated a significant increase in the occurrence of complications with increasing stone complexity. At the end of 1 month, 8 (15.4%) patients had residual stones, while 44 (84.6%) patients were stone-free.

**Figure 2.**
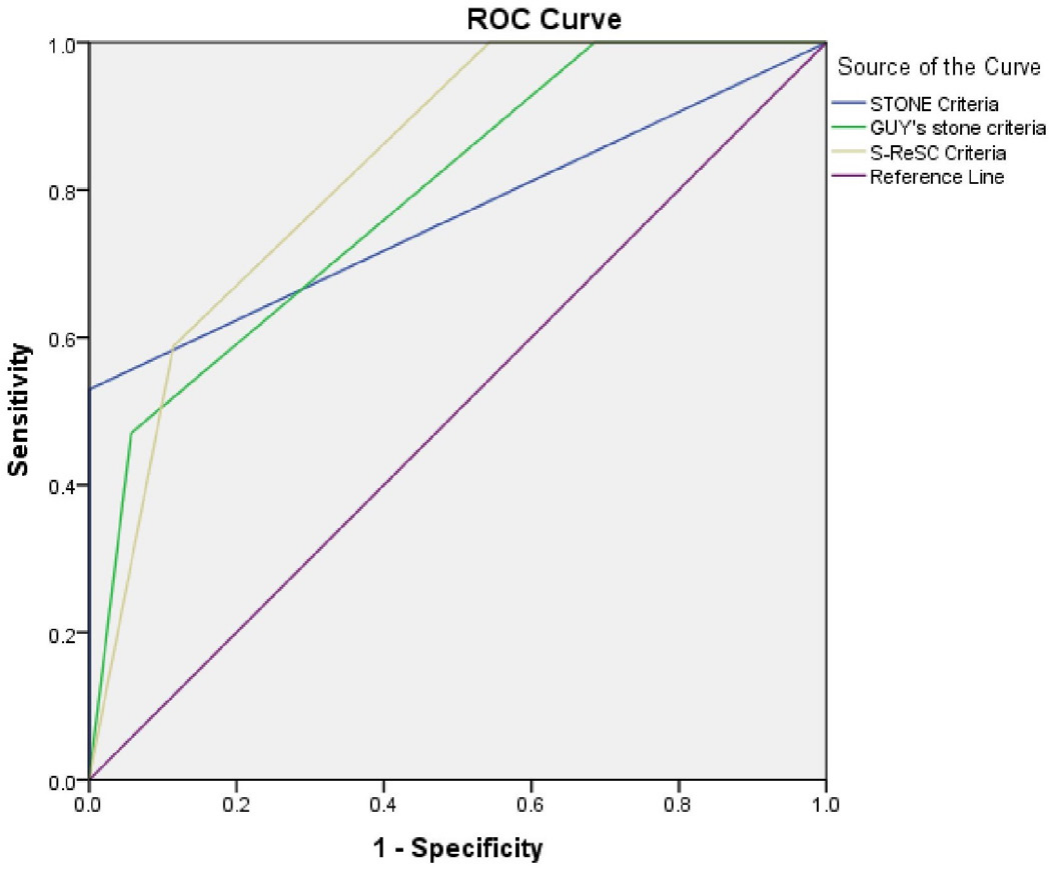
Receiver operating characteristic curve illustrating the predictive accuracy of STONE, Guy’s stone, and S-ReSC stone criteria for determining stone-free status following PCNL.

In the ROC curve analysis presented Table 4 and illustrated in Figure 2, the performance of various scoring systems in predicting stone-free status followingPCNL was evaluated. The AUC for the STONE criteria was found to be 0.765, with a 95% CI ranging from 0.605 to 0.924. In comparison, the Guy’s stone criteria exhibited a slightly higher AUC of 0.790, with a 95% CI extending from 0.662 to 0.918. The S-ReSC scoring system, however, demonstrated the highest AUC among the 3 scoring systems, with a value of 0.831 and a 95% confidence interval from 0.721 to 0.941. The elevated AUC for the S-ReSC scoring system reflects its enhanced accuracy and predictive capability in assessing stone-free status. Despite these findings, it is important to note that pairwise comparisons of the AUCs for these scoring systems, conducted using the DeLong test, did not reveal any statistically significant differences between the STONE, Guy, and S-ReSC criteria (Table 5).

## Discussion

Evaluating staghorn stone complexity using STONE scoring, GUY’s stone score and S-ReSC scoring systems offers valuable insights into their predictive accuracy and their influence on PCNL treatment outcomes. In this study, the STONE criteria classified 61.5% of staghorn stones as low complexity, while 21.2% and 17.3% were categorized as moderate and high complexity, respectively. In contrast, Guy’s Stone score categorized 88.5% of patients as grade III and 11.5% as grade IV stones. The S-ReSC score system similarly identified 84.6% of patients with moderately complex stones and 15.4% with highly complex stones. The discrepancy between these systems highlights their differing approaches to complexity assessment. While the STONE criteria may underestimate complexity, Guy’s Stone score and S-ReSC provide a more detailed classification.

The impact of these classifications on postoperative outcomes is highlighted by the residual stone data. In this study, 32.7% of patients had residual stones, a figure that aligns with the 28% reported by Handa A et al^16^ Jeong CW et al^10^ proposed the S-ReSC score for predicting stone-free rate after PCNL by studying 100 renal stone patients, which reported the overall SFR as 72%, aligning with the findings of current study. In contrast, Kumar U et al^17^ reported a lower residual stone rate of 13.7% following PCNL. The finding from this study that stone complexity directly affects the likelihood of achieving a stone-free status is consistent with the results observed by Kumar U et al.^17^ Specifically, this study demonstrated that both Guy’s Stone score and S-ReSC score systems predicted that all patients with grade IV and high complexity stones, respectively, had residual stones. In contrast, the STONE criteria indicated that 11.1% of patients with high complexity stones were stone-free, highlighting a potential difference in predictive accuracy or a discrepancy in classification severity.

Regarding complications, the Clavien–Dindo classification indicated that 75% of patients had no complications, while 13.5% experienced grade I complications, and 11.5% had grade II complications. Jeong CW et al^10^ also reported a complication rate of 25.2%, with 7.1% of cases classified as grade III. All 3 scoring systems demonstrated a significant increase in complication rates with higher stone complexity. Rathee VS et al^18^ identified a strong correlation of STONE criteria and Guy’s Stone score with complication rates. Similarly, Jeong CW et al^10^ noted a correlation between stone severity and complication rates, although it did not reach statistical significance (*P* = .166). These findings highlight the need for careful assessment of stone complexity to reduce the risk of complications during and after PCNL.

In this study, researchers performed an extensive ROC curve analysis to evaluate the predictive accuracy of various scoring systems for determining stone-free status following PCNL. The S-ReSC scoring system demonstrated the highest AUC at 0.831, which aligns with previous research conducted by Jeong CW et al,^10^ who reported a comparable AUC of 0.853. This high AUC value indicates that the S-ReSC scoring system possesses superior predictive performance compared to other scoring systems, specifically Guy’s Stone criteria and the STONE criteria. In contrast, the AUC for Guy’s Stone criteria in this study was 0.790, and the STONE criteria had an AUC of 0.765. These values suggest that while Guy’s Stone criteria and the STONE criteria have moderate efficacy in evaluating stone complexity and potential treatment outcomes, they do not predict stone-free status as accurately as the S-ReSC scoring system. Furthermore, these findings are consistent with those reported by Handa A et al,^16^ who found AUCs of 0.694 for Guy’s Stone criteria and 0.710 for the STONE criteria, respectively.

These comparative results emphasize that the S-ReSC scoring system stands out as the most precise predictor of achieving a stone-free status post-PCNL. Although Guy’s Stone score and the STONE criteria are useful for assessing stone characteristics and treatment planning, their slightly lower AUCs suggest that they offer reduced predictive accuracy compared to the S-ReSC system. This underscores the value of the S-ReSC scoring system in clinical settings, where the accurate prediction of stone-free status is crucial for optimizing patient outcomes.

The study’s strengths lie in its robust methodology and the thorough examination of 3 distinct scoring systems (STONE, Guy’s Stone, and S-ReSC). By systematically comparing these scoring systems, the study provides valuable insights into their relative effectiveness in predicting outcomes specifically for PCNL monotherapy. This comprehensive comparison is crucial for clinicians as it allows for a better understanding of which scoring system may offer superior predictive value in different clinical scenarios. Moreover, the study’s focus on staghorn stones, recognized for their inherent complexity and difficulty, adds a layer of depth to its findings. Staghorn stones represent a particularly challenging subset of renal stones due to their size and the intricate nature of their treatment. By concentrating on this complex stone type, the study enhances the relevance and applicability of its results to more difficult and demanding cases, thereby offering valuable guidance for managing high-complexity renal stone patients.

However, it is important to acknowledge certain limitations within the study. Firstly, being a single-center study, the findings may have limited generalizability to other clinical settings or geographic locations. Results obtained from a single institution might not fully reflect variations in patient populations or clinical practices at different centers, potentially affecting the broader applicability of the conclusions. Secondly, the relatively short follow-up period could impact the assessment of stone-free status. A brief follow-up duration may not capture all instances of residual stones, as some stones might only become apparent after a longer period. This limitation suggests that the long-term effectiveness of the scoring systems in predicting stone-free status could be influenced by the duration of follow-up and might benefit from extended observation to provide a more comprehensive assessment.

## Conclusions

In conclusion, the preoperative characterization of renal stones plays a crucial role in providing patients with an informed understanding of their likelihood of achieving a stone-free status following PCNL, as well as the potential complications associated with the procedure. Accurate preoperative assessment is essential for optimal patient management and decision-making. Among the STONE, Guy’s stone criteria, and the S-ReSC scoring system evaluated in this study, the S-ReSC system has demonstrated the highest effectiveness in predicting both stone-free rates and complications. This scoring system particularly stands out when dealing with complex cases involving renal staghorn stones, which are known for their challenging nature and higher complication rates. Looking ahead, there is a significant opportunity to enhance the understanding of these scoring systems further. Large-scale, multicenter prospective studies could provide additional insights and validation of the effectiveness of these scoring systems across diverse patient populations and clinical settings. Such studies would help clarify the role of each scoring system in predicting stone-free rates and complications, offering more robust evidence to guide clinical practice.

## Figures and Tables

**Table 1.** Demographic and Clinical Characteristics of Staghorn Calculi Patients

Variable	No. of Patients (n = 52)	Percentage (%)
**1. Age Category**		
21 to 40 years	9	17.3
41 to 60 years	34	65.4
> 60 years	9	17.3
**2. Gender**		
Male	35	67.3
Female	17	32.7
**3. Body Mass Index**		
Normal (18.50 to 24.99 Kg/m^2^)	30	57.7
Overweight (25.00 to 29.99 Kg/m^2^)	20	38.5
Obesity (≥ 30 Kg/m^2^)	2	3.8
**4. Side of the Kidney Involved**		
Right	27	51.9
Left	25	48.1
**5. Presenting Complaints**		
Pain	52	100
Hematuria	13	25
Fever	5	9.6
**6. Stone Size**		
0-399 mm^2^	24	46.1
400-799 mm^2^	16	30.8
800-1599 mm^2^	8	15.4
≥ 1600 mm^2^	4	7.7
**7. Tract Length**		
< 100 mm	51	98.1
≥ 100 mm	1	1.9
**8. Obstruction**		
Yes	26	50
No	27	50
**9. Number of Calyces Involved**		
1-2	34	65.4
3	12	23.1
Staghorn	6	11.5
**10. Essence (HU)**		
≤ 500	5	9.6
501 to 1000	41	78.9
> 1000	6	11.5

HU, Hounsfield units.

**Table 2.** Comparing Stone Free Rate with STONE, Guy’s Stone, and S-ReSC Scoring Systems

Criteria for Staghorn Stone Categorization		Residual Stone Patients (n = 17)		Stone Free Patients (n = 35)		*Χ²* value (*df*)	*P*
		**N**	**%**	**N**	**%**		
STONE criteria	**Low complexity (I)**	1	3.1	31	96.9	33.64 (2)	**< .001; S**
	**Moderate complexity (II)**	8	72.7	3	27.3		
	**High complexity (III)**	8	88.9	1	11.1		
Guy’s Stone criteria	**Grade III**	11	23.9	35	76.1	13.96 (1)	**< .001; S**
	**Grade IV**	6	100	0	0		
S-ReSC criteria	**Moderate complexity (II)**	8	18.6	35	81.4	22.41 (1)	**< .001; S**
	**High complexity (III)**	9	100	0	0		

df, degree of freedom; S, significant; S-,ReSC, Seoul National University Renal Stone Complexity.

**Table 3.** Comparing the Complications with STONE, Guy’s Stone, and S-ReSC Scoring Systems

Criteria for Staghorn Stone Categorization		Complications Based on Clavien–Dindo Classification						*P*
		NIL (n = 39)		STAGE I (n = 7)		STAGE II (n = 6)		
		N	%	N	%	N	%	
STONE criteria	**Low complexity (I)**	32	100	0	0	0	0	**< .001; S**
	**Moderate complexity (II)**	5	45.5	4	36.4	2	18.1	
	**High complexity (III)**	2	22.2	3	33.3	4	44.5	
Guy’s Stone criteria	**Grade III**	39	84.8	4	8.7	3	6.5	**< .001; S**
	**Grade IV**	0	0	3	50	3	50	
S-ReSC criteria	**Moderate complexity (II)**	39	88.6	3	6.8	2	4.6	**< .001; S**
	**High complexity (III)**	0	0	4	50	4	50	

S, significant; S-,ReSC, Seoul National University Renal Stone Complexity.

**Table 4.** Area Under the Curve of the STONE Criteria, Guy’s Stone Criteria, and S-ReSC Criteria in Predicting Stone-Free Rates Following PCNL

Criteria	Area Under Curve	Standard Error	95% Confidence Interval		*P*
			Lower b=Bound	Upper Bound	
STONE Criteria	0.765	0.081	0.605	0.924	.002; S
Guy’s stone criteria	0.790	0.065	0.662	0.918	.001; S
S-ReSC criteria	0.831	0.056	0.721	0.941	< .001; S

S, significant; S-,ReSC, Seoul National University Renal Stone Complexity.

**Table 5.** Pair-Wise Comparison of the Area Under the Curve of the STONE Criteria, Guy’s Stone Criteria, and S-ReSC Criteria by Using the DeLong Test

Comparison of	Difference in AUC	95% Confidence Interval		*P*	Correlation
		Lower Bound	Upper Bound		
STONE criteria and Guy’s stone criteria	−0.025	−0.112	0.062	.569; NS	0.717
STONE criteria and S-ReSC criteria	−0.066	−0.185	0.052	.274; NS	0.434
Guy’s Stone criteria and S-ReSC criteria	−0.041	−0.135	0.052	.388; NS	0.572
*P*-value of overall comparison of 3 scoring systems = .543; NS					

AUC, area under the curve; NS, not significant; S-,ReSC, Seoul National University Renal Stone Complexity.

## Data Availability

The data that support the findings of this study are available on request from the corresponding author.
